# Protein inhibitor of activated STAT 4 (PIAS4) regulates pro-inflammatory transcription in hepatocytes by repressing SIRT1

**DOI:** 10.18632/oncotarget.9864

**Published:** 2016-06-06

**Authors:** Lina Sun, Zhiwen Fan, Junliang Chen, Wenfang Tian, Min Li, Huihui Xu, Xiaoyan Wu, Mingming Fang, Jun Xia, Yong Xu

**Affiliations:** ^1^ State Key Laboratory of Reproductive Medicine, Department of Pathophysiology, Key Laboratory of Cardiovascular Disease and Molecular Intervention, Nanjing Medical University, Nanjing, China; ^2^ Department of Pathology and Pathophysiology, School of Biology and Basic Medical Sciences, Soochow University, Suzhou, China; ^3^ Department of Pathology, Nanjing Drum Tower Hospital, the Affiliated Hospital of Nanjing University Medical School, Nanjing, China; ^4^ Department of Pathophysiology, School of Basic Medical Sciences, Jiangnan University, Wuxi, China; ^5^ Department of Nursing, Jiangsu Jiankang Vocational University, Nanjing, China; ^6^ Department of Respiratory Medicine, Jiangsu Province Hospital of Traditional Chinese Medicine, Nanjing, China

**Keywords:** transcriptional regulation, inflammation, PIAS4, SIRT1, NF-κB, hepatocyte, Pathology Section

## Abstract

Excessive nutrition promotes the pathogenesis of non-alcoholic steatohepatitis (NASH), characterized by the accumulation of pro-inflammation mediators in the liver. In the present study we investigated the regulation of pro-inflammatory transcription in hepatocytes by protein inhibitor of activated STAT 4 (PIAS4) in this process and the underlying mechanisms. We report that expression of the class III deacetylase SIRT1 was down-regulated in the livers of NASH mice accompanied by a simultaneous increase in the expression and binding activity of PIAS4. Exposure to high glucose stimulated the expression PIAS4 in cultured hepatocytes paralleling SIRT1 repression. Estrogen, a known NASH-protective hormone, ameliorated SIRT1 trans-repression by targeting PIAS4. Over-expression of PIAS4 enhanced, while PIAS4 knockdown alleviated, repression of SIRT1 transcription by high glucose. Lentiviral delivery of short hairpin RNA (shRNA) targeting PIAS4 attenuated hepatic inflammation in NASH mice by restoring SIRT1 expression. Mechanistically, PIAS4 promoted NF-κB-mediated pro-inflammatory transcription in a SIRT1 dependent manner. In conclusion, our study indicates that PIAS4 mediated SIRT1 repression in response to nutrient surplus contributes to the pathogenesis of NASH. Therefore, targeting PIAS4 might provide novel therapeutic strategies in the intervention of NASH.

## INTRODUCTION

Nutrient surplus induces a pro-inflammatory niche and inflicts insults in different types of cells and organs. This scenario constitutes the major mechanism underlying the pathogenesis of non-alcoholic steatohepatitis (NASH) characterized by numerous inflammatory infiltrates in the liver eventually transitioning into cirrhosis and hepatocellular carcinoma [1]. In patients with NASH, expression of inflammatory and immune response genes, such as IL-1β, IL-6, and MCP-1, was significantly up-regulated in the liver in comparison to those with simple steatosis [2]. Furthermore, persistent inflammation in NASH patients are consistent with a poor prognosis [3]. Therefore, a clear understanding of the molecular pathways dictating the transcriptional regulation of pro-inflammatory mediators will aid the development of novel therapeutics for the intervention of NASH.

In mammals, expression of pro-inflammatory is programmed by a handful of evolutionarily conserved transcription factors such as NF-κB [4]. The activity of these factors and consequently the intensity of pro-inflammatory transcription are regulated at multiple levels. For instance, NF-κB proteins are subject to post-translational modifications (e.g., phosphorylation, methylation, and acetylation), which can modulate its subcellular localization, interaction with co-factors, and affinity for target genes [5]. Specifically, deacetylation of NF-κB/p65 by SIRT1 suppresses its activity and dampens pro-inflammatory transcription [6]. Therefore, SIRT1 stands at a critical juncture of cellular inflammatory responses by modifying and hence fine-tuning the activities of key transcription factors.

Mounting evidence suggests that SIRT1, a class III NAD-dependent deacetylase, plays diverse roles in a number of cardiovascular and metabolic diseases including NASH [7]. SIRT1 activation by agonists confers protection against NASH in mice [8, 9]. In contrast, liver-specific deletion of SIRT1 promotes hepatic inflammation/fibrosis and exacerbates NASH in mice [10]. In addition, SIRT1 expression is down-regulated in the liver in both animal models of NASH and in NASH patients [11, 12]. These investigations collectively argue for enhancing SIRT1 expression and/or activity as a means of combating NASH. The mechanism whereby SIRT1 expression is down-regulated in the liver in the context of NASH is not completely understood.

Previously, our laboratory has identified a SUMOylation dependent pathway wherein the SUMO E3 ligase PIASy (PIAS4) represses SIRT1 expression in response to hypoxia in cancer cells [13, 14]. Here we demonstrate that PIAS4 mediates SIRT1 repression *in vitro* and *in vivo* in response to nutrient surplus. As such, our data provide novel insights into the development of interventional strategies for NASH.

## RESULTS

### PIAS4 activation parallels SIRT1 repression *in vivo*

**Figure 1:**
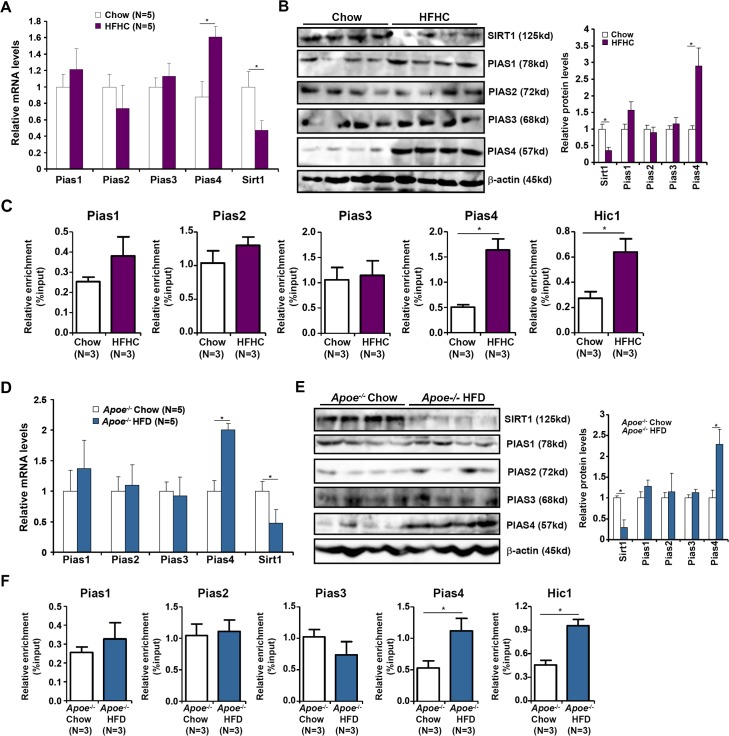
Increased PIAS4 expression accompanies repression of SIRT1 *in vivo* **A.**-**C.** C57/BL6 mice were fed on a high-fat and high-carbohydrate (HFHC) diet or a control diet (chow) for 16 weeks. mRNA (A) and protein (B) levels were measured by qPCR and Western. (C) Binding of PIAS proteins and HIC1 to the SIRT1 promoter was evaluated by ChIP. **D.**-**F.** *Apoe*^−/−^mice were fed on a high-fat diet (HFD) or a control diet (chow) for 8 weeks. mRNA (D) and protein (E) levels were measured by qPCR and Western. (F) Binding of PIAS proteins and HIC1 to the SIRT1 promoter was evaluated by ChIP.

To probe the relevance of PIAS4 in NASH pathogenesis, we first examined the expression of PIAS4 in different experimental NASH models in mice. In the first model, C57/BL6 mice were induced to develop NASH by a high-fat, high-carbohydrate (HFHC) diet plus fructose-containing drinking water [15]. Compared to mice fed on a control diet, NASH mice exhibited increased expression of PIAS4 in the liver (Figure 1A, 1B); other members of the PIAS family including PIAS1, PIAS2, and PIAS3 were not significantly altered. Consistent with our previous finding that PIAS4 represses SIRT1 transcription in cancer cells [13, 14], we observed a decrease in SIRT1 levels in the livers of NASH mice compared to the control mice with concomitant increase in the binding of PIAS4 as well as HIC1-a known SIRT1 repressor and PIAS4 substrate-on the SIRT1 promoter region (Figure 1C). Similar observations were made in a second model in which *Apoe*^−/−^ mice were fed a western diet (HFD) for 8 weeks (Figure 1D-1F). Therefore, PIAS4 might be responsible for SIRT1 trans-repression during NASH pathogenesis.

### PIAS4 activation parallels SIRT1 repression *in vitro*

**Figure 2:**
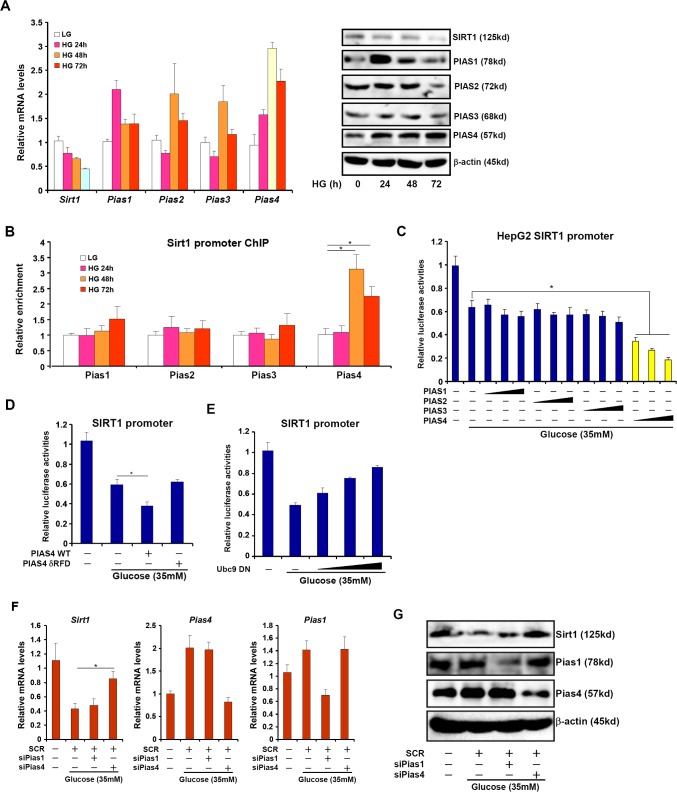
Increased PIAS4 expression accompanies repression of SIRT1 in cultured hepatocyte **A.**, **B.** Primary mouse hepatocytes were treated with glucose (35mM) and harvested at indicated time points. mRNA and protein levels were measured by qPCR and Western (A). PIAS binding to the SIRT1 promoter was examined by ChIP (B). **C.** A SIRT1 promoter-luciferase construct was transfected into HepG2 cells along with indicated PIAS expression constructs followed by treatment with high glucose for 24 hours. **D.** A SIRT1 promoter-luciferase construct was transfected into HepG2 cells along with wild type (WT) or enzyme deficient (δRFD) PIAS4 construct followed by treatment with high glucose for 24 hours. **E.** A SIRT1 promoter-luciferase construct was transfected into HepG2 cells along with increasing amounts of Ubc9 DN construct followed by treatment with high glucose for 24 hours. Data are expressed as relative luciferase activities. **F.**, **G.** Primary hepatocytes were transfected with indicated siRNAs followed by treatment with glucose. mRNA (F) and protein (G) levels of SIRT1 were measured by qPCR and Western.

Next, we asked whether our model, i.e., PIAS4 represses SIRT1 transcription in the liver, was operative in cultured hepatocyte in response to nutrient surplus. Indeed, exposure to high glucose (35mM) led to a decrease in SIRT1 expression while simultaneously stimulating PIAS4 expression in both primary mouse hepatocytes (Figure 2A) and immortalized hepatocellular carcinoma cells (HepG2, Figure S1A, S1B). High glucose also promoted the binding of PIAS4 on the SIRT1 promoter in line with its role as a repressor of SIRT1 transcription (Figure 2B, S1C). In addition, PIAS4, instead of other PIAS proteins, synergized with high glucose to repress SIRT1 promoter activities (Figure 2C). Furthermore, a PIAS4 mutant lacking the enzyme domain (δRFD) failed to enhance SIRT1 promoter repression by glucose (Figure 2D). On the contrary, over-expression of a dominant negative form of Ubc9 (Ubc9 DN), the enzyme that precedes PIAS4 in catalyzing the SUMO transfer reaction, reversed the repression of SIRT1 promoter by high glucose (Figure 2E). Finally, siRNA-mediated depletion of PIAS4 restored SIRT1 expression despite the presence of high glucose in both primary hepatocytes (Figure 2F, 2G) and HepG2 cells (Figure S2). Of note, 17β-estradiol with a known property of antagonizing NASH [16, 17] could block the induction of PIAS4 expression and SIRT1 promoter binding by high glucose (Figure S3). Together, these data further allude to the possibility that PIAS4 might play a role in SIRT1 repression in response to excessive nutrient in the liver.

### PIAS4 knockdown alleviates hepatic inflammation in NASH mice

**Figure 3:**
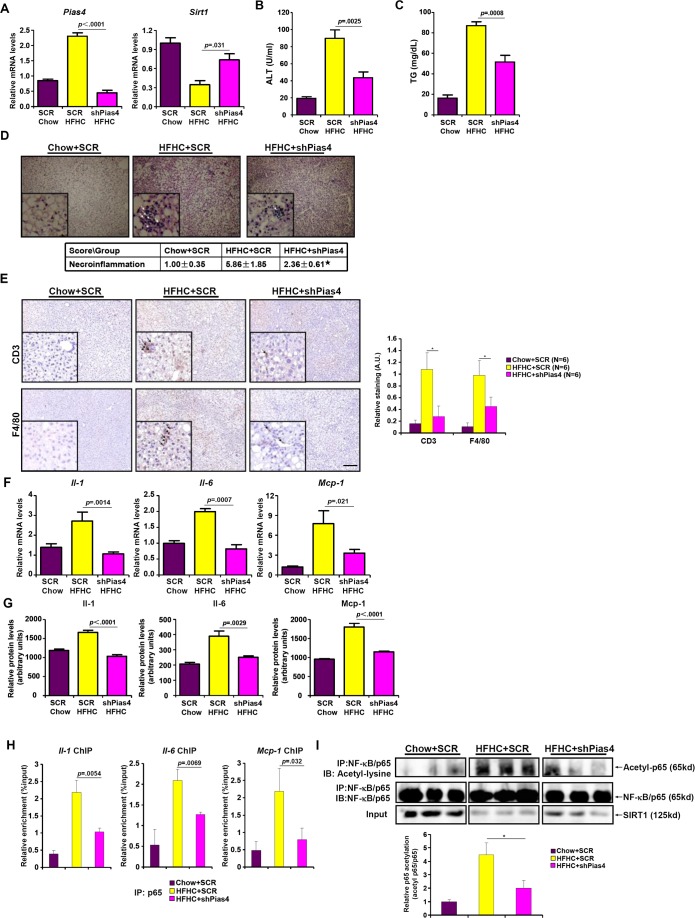
PIAS4 depletion attenuates hepatic inflammation in mouse models of NASH Male C57/BL6 mice were fed with indicated diets for 16 weeks. Silencing of PIAS4 was mediated by lentivirus as described under Methods. **A.** Expression of Pias4 and Sirt1 was measured by qPCR. **B.**, **C.** Levels of ALT (A) and TG (B) were measured by ELSA. *N* = 6 mice for each group. **D.** H&E staining was performed as described under Methods. *, *p* < .05 **E.** Immunohistochemistry was performed using paraffin embedded liver sections with anti-CD3 and anti-F4/80. Scale bar, 50μm. **F.**, **G.** Expression of pro-inflammatory mediators were measured by qPCR or ELISA. *N* = 6 mice for each group. **H.** Binding of NF-κB/p65 to pro-inflammatory genes was determined by ChIP using liver homogenates. *N* = 3 mice for each group. **I.** Acetylation levels of NF-κB/p65 in the liver were determined by Western.

We next made an attempt to further validate the role of PIAS4 *in vivo* in the pathogenesis of NASH in mice. Injection of lentivirus carrying interfering RNA against PIAS4 (shPias4) into the NASH mice induced by the HFHC diet resulted in ~80% decrease in PIAS4 expression in the liver compared to the NASH mice injected with an empty vector (SCR) as measured by qPCR (Figure 3A); PIAS4 silencing led to a significant up-regulation of SIRT1 expression in the liver (Figure 3A). Of note, PIAS4 silencing did not significantly alter global SUMOylation levels in the liver (Figure S4). Meanwhile, PIAS4 knockdown partially corrected the impairment of metabolic profiles associated with steatohepatitis as evidenced by attenuation of plasma ALT (Figure 3B) and liver TG (Figure 3C) levels. H&E staining revealed significantly fewer necroinflammation nodules in mice receiving shPias4 virus when compared to the control mice (Figure 3D). Meanwhile, infiltration of pro-inflammatory immune cells in the liver including CD3+ T lymphocytes and F4/80+ macrophages was down-regulated in mice following PIAS4 knockdown (Figure 3E). As a result, hepatic inflammation was ameliorated as demonstrated by the expression levels of pro-inflammatory mediators (Figure 3F, 3G). Consistently, we observed suppressed binding of NF-κB/p65 to its target promoters (Figure 3H), which was probably due to SIRT1-mediated deacetylation of NF-κB/p65 (Figure 3I). Collectively, this line of evidence suggests that PIAS4 might regulate hepatic inflammation to promote NASH in mice likely through repressing SIRT1 expression.

### PIAS4 stimulates pro-inflammatory transcription in response to high glucose stimulation

**Figure 4:**
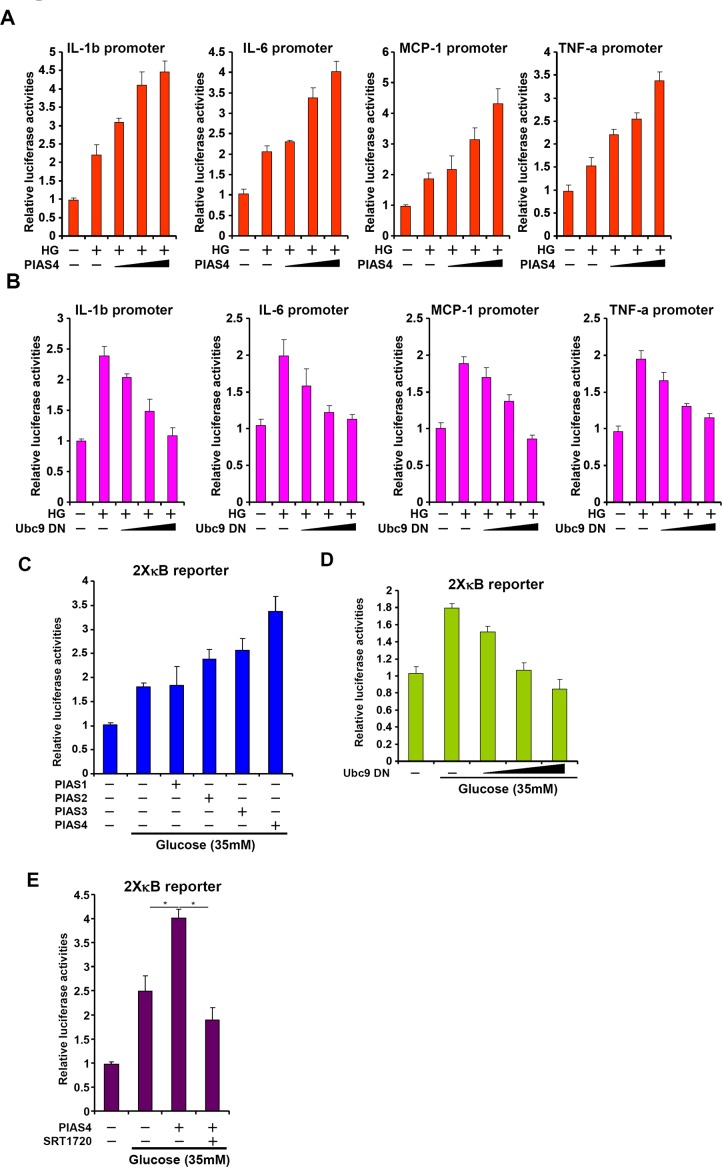
PIAS4 stimulates pro-inflammatory transcription in response to high glucose stimulation in culture hepatocyte **A.** Indicated promoter luciferase constructs were transfected into HepG2 cells with or without PIAS4 followed by treatment with high glucose for 24 hours. **B.** Indicated promoter luciferase constructs were transfected into HepG2 cells with or without Ubc9 DN followed by treatment with high glucose for 24 hours. **C.** An NF-κB reporter was transfected into HepG2 cells along with indicated expression constructs followed by treatment with glucose. **D.** An NF-κB reporter was transfected into HepG2 cells with or without Ubc9 DN followed by treatment with glucose. **E.** An NF-κB reporter was transfected into HepG2 cells along with indicated expression constructs followed by treatment with glucose and/or SRT1720. Data are expressed as relative luciferase activities.

Having established a potential role for PIAS4 in regulating hepatic inflammation during NASH pathogenesis, we next probed the underlying mechanism in cultured hepatocytes. We first examined whether PIAS4 could promote pro-inflammatory transcription in response to high glucose. Over-expression of PIAS4 in HepG2 cells enhanced the activation of several NF-κB target promoters including IL-1, IL-6, MCP-1, and TNF-a when exposed to high glucose (Figure 4A). By comparison, over-expression of Ubc9 DN dose-dependently suppressed transactivation of pro-inflammatory gene promoters in cells treated with high glucose (Figure 4B). More importantly, PIAS4, but not other PIAS proteins, enhanced the activation of a generic κB reporter by high glucose, indicating that PIAS4 could be a *de novo* regulator of NF-κB-dependent pro-inflammatory transcription. Consistently, Ubc9 DN abrogated transactivation of the κB reporter by high glucose (Figure 4D). Together, these data collectively suggest that PIAS4 could promote hepatic pro-inflammatory transcription in response to nutrition surplus.

### PIAS4 regulates inflammatory response in cultured hepatocyte by repressing SIRT1 expression

**Figure 5:**
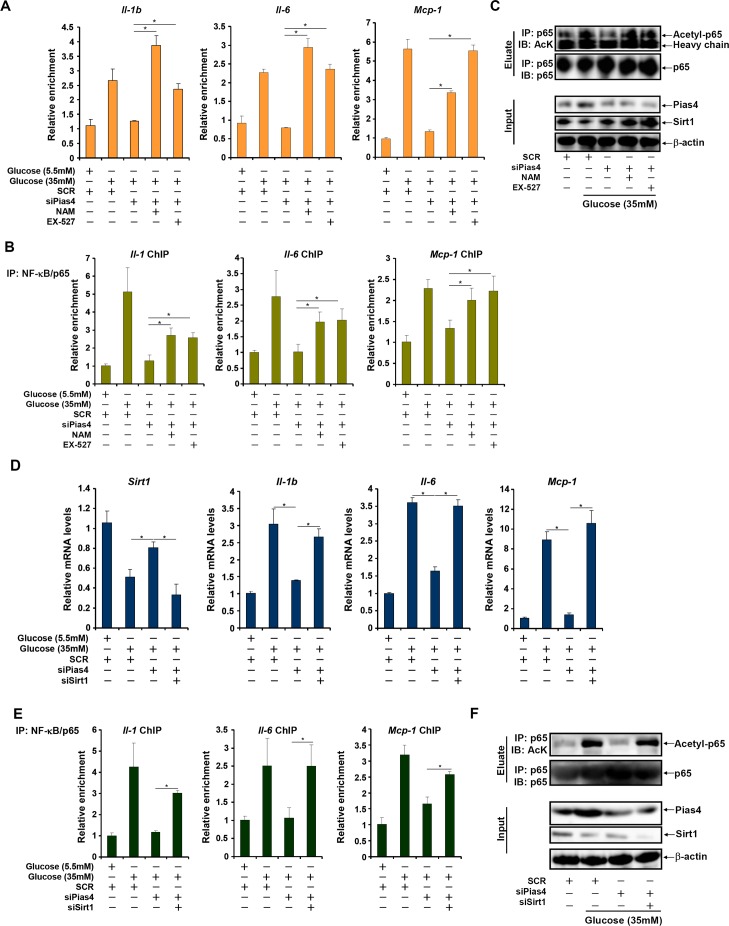
PIAS4 regulates inflammatory response by repressing SIRT1 expression **A.**-**C.** Primary hepatocytes were transfected with indicated siRNAs followed by treatment with glucose, NAM, and/or EX-527. mRNA levels of pro-inflammatory mediators were measured by qPCR (A). Binding of p65 to pro-inflammatory genes was determined by ChIP (B). (C) Whole cell lysates were immunoprecipitated with anti-p65 and the precipitated immune complex (eluate) was separated by SDS-PAGE gel electrophoresis. Western blotting was performed with indicated antibodies. 10% of the starting material was included as input. **D.**-**F.** Primary hepatocytes were transfected with indicated siRNAs followed by treatment with glucose. mRNA levels of pro-inflammatory mediators were measured by qPCR (D). Binding of p65 to pro-inflammatory genes was determined by ChIP (E). (F) Whole cell lysates were immunoprecipitated with anti-p65 and the precipitated immune complex (eluate) was separated by SDS-PAGE gel electrophoresis. Western blotting was performed with indicated antibodies. 10% of the starting material was included as input.

SIRT1 activation by a specific agonist SRT1720 blocked PIAS4-induced potentiation of NF-κB activity, indicating that PIAS4 might regulate NF-κB-dependent inflammation through modulating SIRT1 expression (Figure 4E). Therefore we asked whether the ability of PIAS4 to promote pro-inflammatory transcription relies on SIRT1. To this end, we performed the following experiments. We found that while PIAS4 knockdown alleviated high glucose-induced production of pro-inflammatory mediators, treatment of two different SIRT1 antagonists, nicotinamide (NAM) and EX-527, completely abrogated this effect (Figure 5A and Figure S5A). In parallel, PIAS4 knockdown alone reduced the binding of NF-κB/p65 on the promoters of pro-inflammatory genes, but pre-treatment with either NAM or EX-527 restored p65 binding (Figure 5B and Figure S5B). Similarly, simultaneous knockdown of PIAS4 and SIRT1 restored reversed the effect of PIAS silencing by bringing up the synthesis of pro-inflammatory mediators (Figure 5D and Figure S6A) and NF-κB target binding (Figure 5E and Figure S6B). Consistent with these observations, PIAS4 up-regulated NF-κB acetylation in a SIRT1-dependent manner in hepatocytes (Figure 5C, 5F). Together, these data confirm that PIAS4 regulates the inflammatory response in cultured hepatocyte by repressing SIRT1 expression.

## DISCUSSION

**Figure 6:**
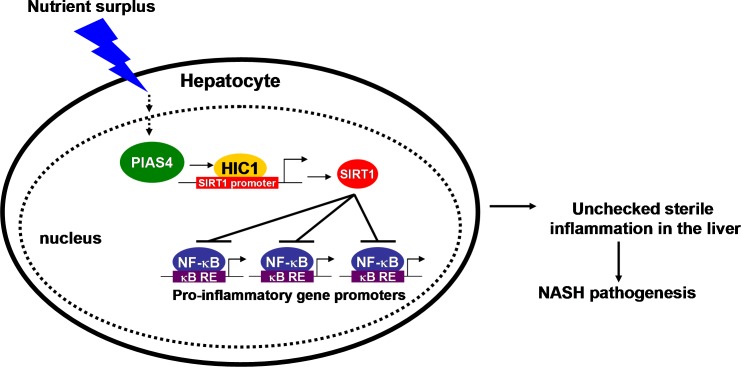
A schematic model depicting the potential role of PIAS4 in NASH pathogenesis In response to nutrition surplus in hepatocytes, PIAS4 up-regulation results in SIRT1 repression and NF-κB hyper-acetylation. Activated NF-κB binds to its target promoters and drives the transcription of pro-inflammatory genes. Consequently, accumulation of pro-inflammatory mediators in the liver contributes to NASH.

Owing much to changes in life style and diets in the past quarter century, NASH has been become a global health risk. A hallmark event in the pathogenesis of NASH is augmented hepatic inflammation leading to irreversible end-stage liver disease (e.g., cirrhosis and hepatocellular carcinoma). Sirtuins, particularly SIRT1, have been shown to afford protective effects in the context of NASH by limiting hepatic inflammation [12, 18, 19]. Conversely, SIRT1 expression and activity are down-regulated in the liver as NASH progresses [11, 12]. Our data as summarized here provide a novel mechanism that links nutrient surplus to SIRT1 repression and present PIAS4 as a potential druggable target for NASH intervention (Figure 6).

NASH is primarily a hepatic pathology in which excessive energy input or deficient energy expenditure helps build up a pro-inflammatory microenvironment leading to disease progression. We demonstrate for the first time that PIAS4 expression and activity alter in response to energy input in mouse livers and in cultured hepatocytes. This is consistent with the notion that protein post-translational modification (PTM) enzymes participate in cellular metabolic processes by sensing energy influx/efflux [20–22]. Of note, a proteomic screening has identified over 100 different SUMOylated proteins including PIAS4 itself in cells in response to transient oxygen/glucose deprivation [23]. Previous studies have shown that PIAS4 can be activated by hypoxia, which is a critical factor in regulating energy metabolism, in cancer cells [13, 14, 24, 25]. These separate pieces of evidence collectively suggest that PIAS4 might be an intracellular energy sensor in the development of human pathologies. It is not entirely clear how PIAS4 represses SIRT1 in hepatocytes in the context of NASH pathogenesis since PIAS4 knockdown did not affect global SUMOylation levels in the liver indicating PIAS4 could function through individual transcription factors (Figure S4). It appears that HIC1, a known PIAS4 substrate that represses SIRT1 transcription in a SUMOylation-dependent manner [26], binds to the SIRT1 promoter with higher affinity in the livers of NASH mice (Figure 1C and Figure 1F). HIC1 expression levels have shown to correlate with hepatocarcinogenesis [27, 28], but its role in NASH has yet to be explored. The availability of the HIC1-Flox strain makes it possible to delete HIC1 specifically in the liver and then investigate its role in NASH pathogenesis [29]. Further investigations are warranted to pin down the list of PIAS4 substrates relevant to the pathogenesis of NASH.

SIRT1 mediated suppression of NF-κB dependent pro-inflammatory transcription, via lysine deacetylation of p65, is considered a central mechanism underlying its protective roles in a range of human diseases including atherosclerosis, stroke, and diabetes [30–32]. We have confirmed that in the livers of NASH mice, SIRT1 activity is decreased while simultaneously NF-κB activity is increased when compared to control mice. In addition, we also demonstrate that PIAS4 silencing restores SIRT1 expression but dampens NF-κB acetylation and activity. Therefore, PIAS4 dependent SIRT1 repression leads to NF-κB liberation and accumulation of hepatic inflammation in NASH pathogenesis. This does not rule out the possibility, however, that PIAS4 might directly regulate hepatic inflammation. PIAS4, for instance, has been found to SUMOylate NEMO, an upstream activator for NF-κB, in response to genotoxic stress [33]. PIAS4 is also known to be able to directly bind to NF-κB although the consequences of this interaction are not clear [34]. It would be of great interest to delineate both the SIRT1-dependent and SIRT1-independent roles for PIAS4 in modulating pro-inflammatory transcription in hepatocyte in NASH pathogenesis.

A key issue is left unaddressed by the current report. Although we have demonstrated a role of PIAS4 in hepatocyte, it remains elusive whether there is a communication between different liver cells fueled by PIAS4-mediated transcriptional program. Recently, it has been reported that within the liver, different cell types (e.g., sinusoidal endothelial cells and hepatic stellate cells) may forge extensive dialogues to maintain homeostasis; once this communication is disrupted, liver pathologies ensue [35, 36]. Tissue-specific conditional PIAS4 deficient mice would help resolve this issue.

We show here that PIAS4, but not other PIAS family members, was specifically elevated in different mouse models of NASH and in glucose-stimulated hepatocytes, indicating that there is a functional non-redundancy among PIAS proteins at least in the context of NASH pathogenesis. Of note, a recent report echoes our finding that PIAS proteins play important roles in regulating metabolic inflammation. Liu et al examined expression patterns of PIAS proteins in adipose tissues in diabetic mice [37]; while PIAS1 levels was down-regulated, PIAS4 expression was up-regulated in *db*/*db* mice compared to wild type littermates. In addition, adenovirus-mediated PIAS1 over-expression alleviated inflammation in adipocytes although it remained untested whether PIAS4 depletion would have a similar effect. Because PIAS proteins all rely on the same ring-finger domain (RFD) to regulate pathobiological processes (Figure 2D), this discrepancy-PIAS4 apparently promoting inflammation in the liver while PIAS1 suppressing inflammation in adipose tissue-presents a challenge in screening for and/or delivering small-molecule compound to modulate PIAS activity as a therapeutic solution. Recent advances in molecular docking-based, computer-aided drug design and material/surface science will likely offer help in this matter [38].

In summary, we provide evidence that PIAS4 represses SIRT1 expression in response to excessive energy input and that PIAS4 silencing confers beneficial effects in mice. Future studies should aim at clarifying the cell-specific and substrate-specific roles for PIAS4 thereby rendering targeting PIAS4 in the treatment of NASH a real possibility.

## MATERIALS AND METHODS

### Cell culture, plasmids and transfection

Primary hepatocytes were isolated from C57/BL6 mice as previously described [39]. Immortalized human hepatic carcinoma cells HepG2 (ATCC) were maintained according to vendors' recommendations. The NF-κB reporter construct, promoter luciferase constructs for IL-1b, IL-6, MCP-1, TNF-a, and expression constructs as well as small interfering RNA (siRNA) sequences have been described previously [13, 14, 40, 41]. Transient transfections were performed with Lipofectamine 2000 (Invitrogen). Luciferase activities were assayed using a luciferase reporter assay system (Promega).

### Animals

All animal protocols were approved by the NJMU Intramural Ethic Committee on Animal Studies. To induce steatohepatitis, 8 week-old male C57/BL6 mice were fed a high fat high carbohydrate (HFHC) diet (D12492, Research Diets) for 16 consecutive weeks [15]. Alternatively, 6-8 week-old male Apoe−/− mice (Jackson Laboratory) were fed with a high fat diet (D12079, Research Diets) for 12 weeks. Blood triglyceride (TG) and alanine aminotransferase (ALT) levels were measured as described before [42]. In certain experiments, these mice were injected via tail vein purified lentiviral particles (1×10^9^ MOI) that carry short hairpin RNA (shRNA) targeting PIAS4 (GTGCTGTACGGGAAGTACTT) or scrambled shRNA (SCR) every 10 days for the duration of the experiments.

### Protein extraction and western blot

Tissue and cell lysates were obtained as previously described [43]. Western blot analyses were performed with anti-PIAS1, anti-PIAS2, anti-PIAS3, anti-p65, anti-RNA polymerase II (Santa Cruz), anti-PIAS4 (Sigma), anti-acetyl lysine (Cell Signaling Tech), and anti-SIRT1 (Abcam) antibodies.

### Chromatin immunoprecipitation (ChIP)

ChIP assays were performed essentially as described before [44] with anti-PIAS1, anti-PIAS2, anti-PIAS3, anti-PIAS4, anti-HIC1, and anti-p65 (Santa Cruz). Precipitated genomic DNA was amplified by real-time PCR with primers as previously described [13, 39, 41].

### Histology

Histological analyses were performed essentially as described before [43]. Briefly, paraffin sections were stained with hematoxylin and eosin (Sigma). Parallel sections were stained for cell surface markers.

### Enzyme-linked immune absorbance assay (ELISA)

Supernatants containing pro-inflammatory mediators were collected from cultured hepatocyte or liver homogenates and ELISA was performed to measure IL-1, IL-6, and MCP-1 using commercially available kits (R&D).

### Statistical analysis

Data are presented as mean±SD. For experiments concerning multiple groups, one-way ANOVA with post-hoc Scheffe analyses were performed to evaluate the differences. The differences between two (control and experimental) groups were determined by two-sided, unpaired Student's t-test. *p* values smaller than .05 are considered significant. For the *in vivo* experiments, specific *p* values are spelled out. All the *in vitro* experiments were repeated at least three times with a power analysis showing β>80.

## SUPPLEMENTAL MATERIAL


