# Supplementary material for: Protein inhibitor of activated STAT 4 (PIAS4) regulates pro-inflammatory transcription in hepatocytes by repressing SIRT1
Source: Oncotarget. 2016 Jun 6;7(28):42892–903. doi: 10.18632/oncotarget.9864 (PMC5189995; doi:10.18632/oncotarget.9864)
Supplement: Supplementary file 1 [file oncotarget-07-42892-s001.pdf]

Protein inhibitor of activated STAT 4 (PIAS4) regulates pro-inflammatory transcription in hepatocytes by repressing SIRT1

Supplemental material

A

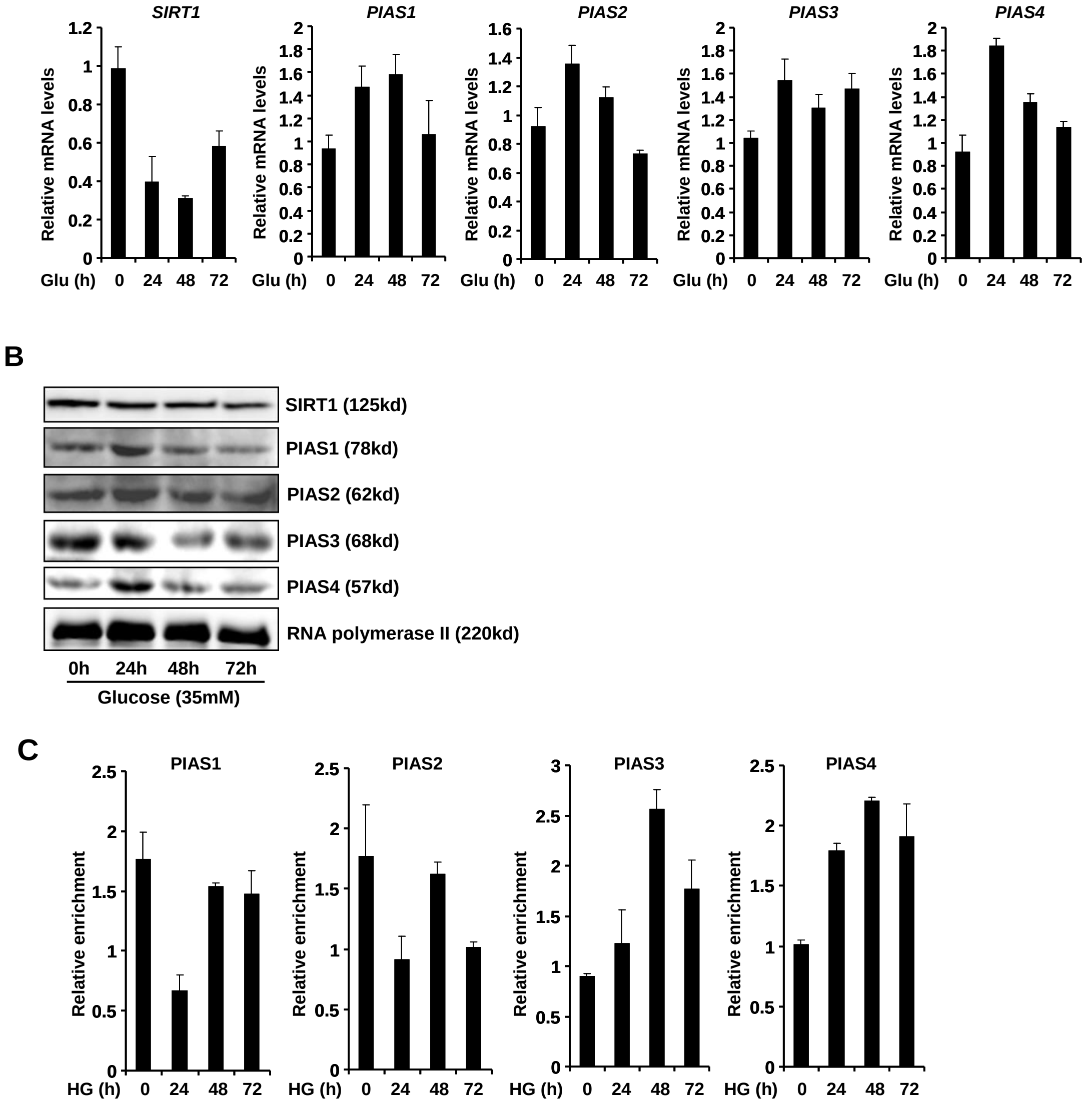

Fig.S1: (**A-C**) HepG2 cells treated with glucose (35mM) and harvested at indicated time points. mRNA (A) and protein (B) levels were measured by qPCR and Western. PIAS binding to the SIRT1 promoter was examined by ChIP (C).

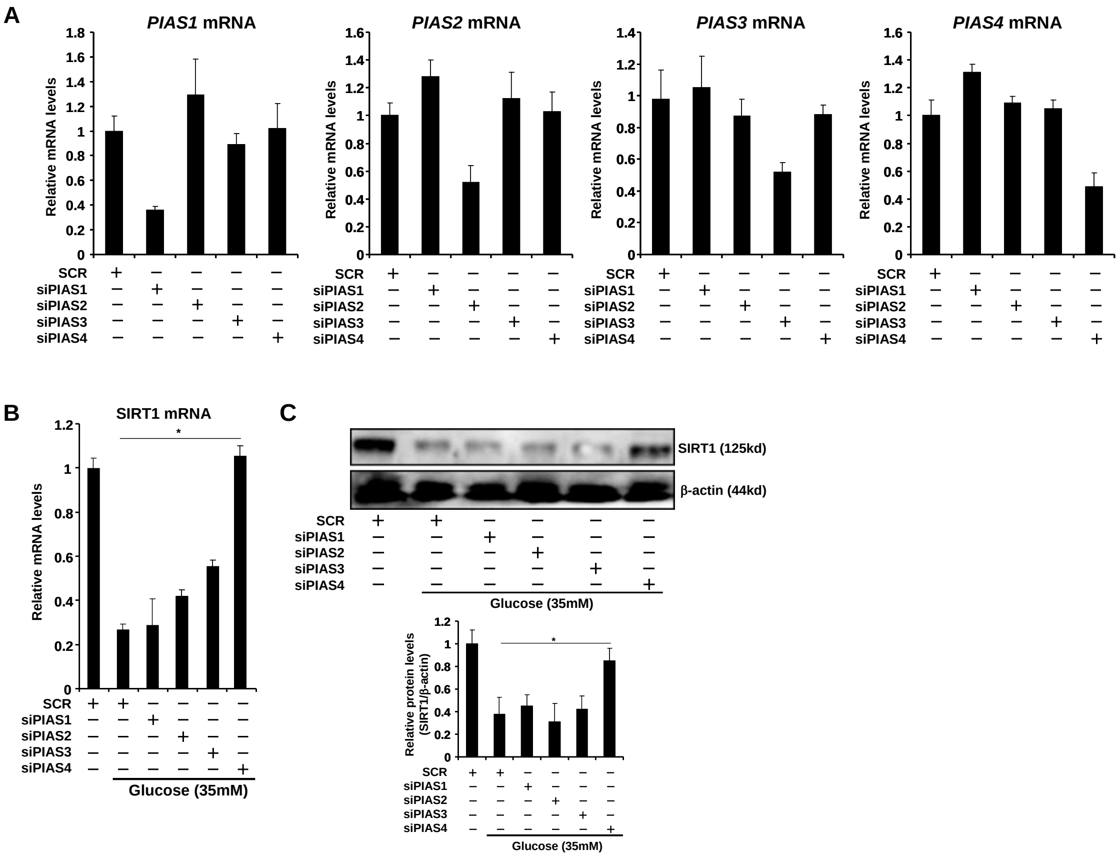

Fig.S2: (**A**) HepG2 cells were transfected with indicated siRNAs. Expression levels of PIAS proteins were measured by qPCR. (**B, C**) HepG2 cells were transfected with indicated siRNAs followed by with glucose. Expression levels of SIRT1 were measured by qPCR and Western.

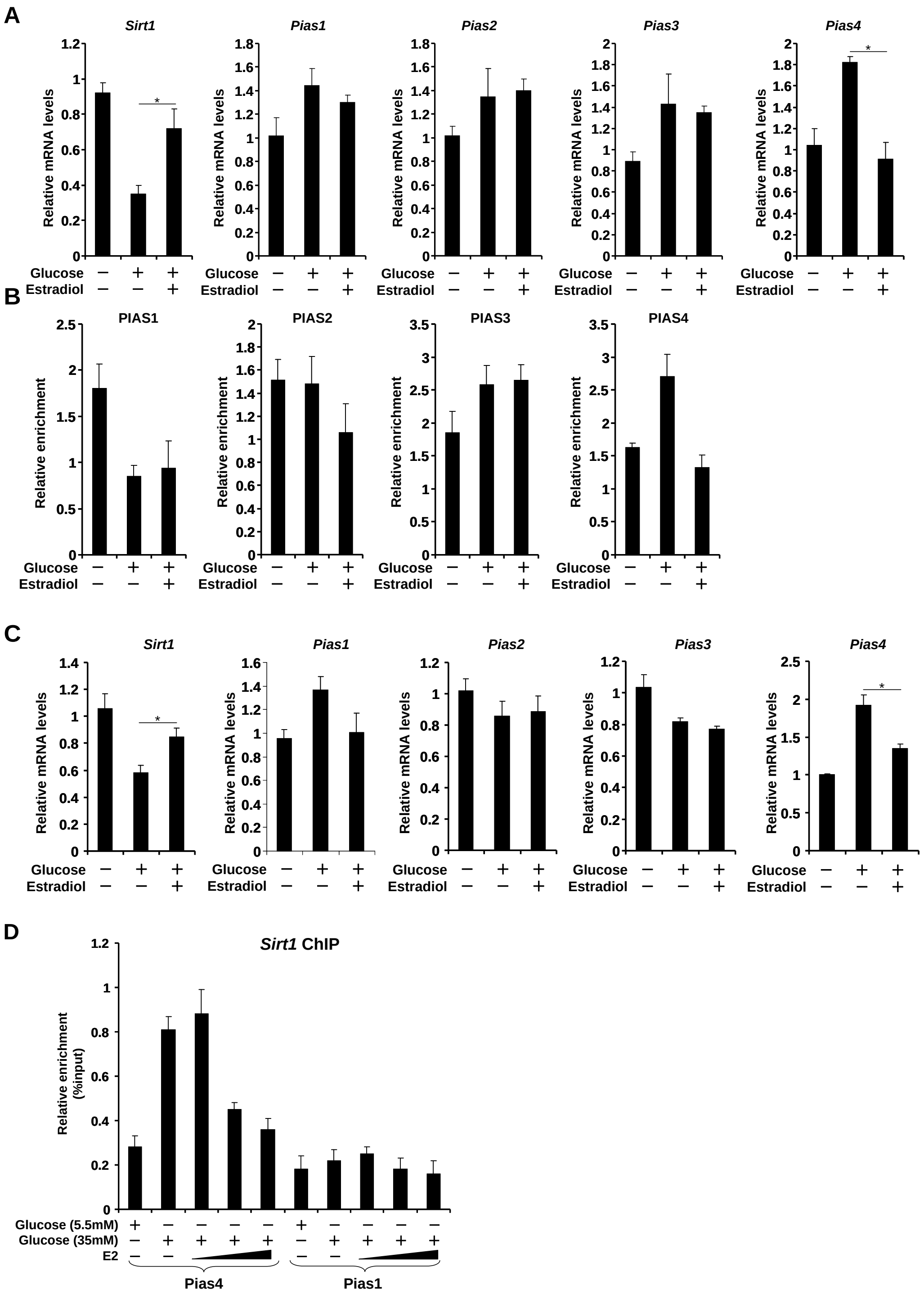

Fig.S3: (**A, B**) HepG2 cells were treated with glucose and/or estradiol for 24 hours. Expression of SIRT1 and PIAS proteins was measured by qPCR (A). Binding of PIAS proteins to the SIRT1 promoter was measured by ChIP (B). (**C, D**) HSC-T6 cells were treated with glucose and/or estradiol for 24 hours. Expression of SIRT1 and PIAS proteins was measured by qPCR (C). Binding of PIAS proteins to the SIRT1 promoter was measured by ChIP (D).

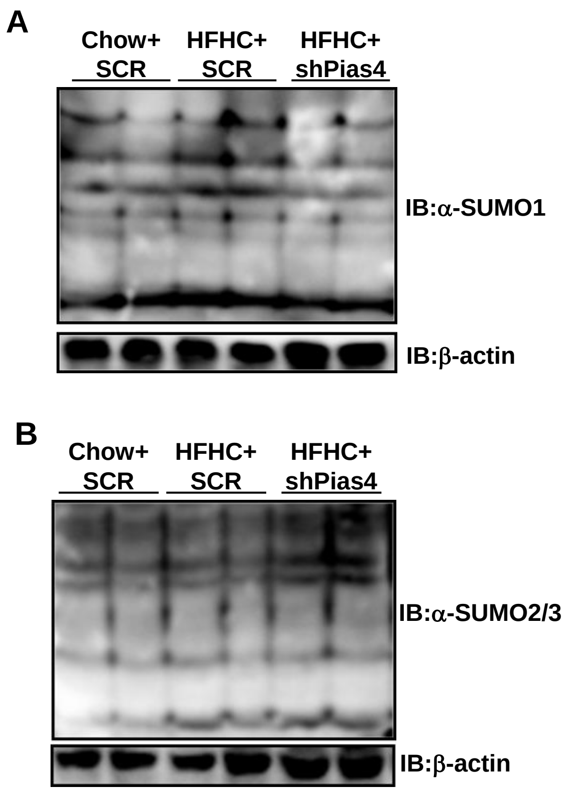

Fig.S4: C57/BL6 mice were fed with indicated diets for 16 weeks. Silencing of PIAS4 was mediated by lentivirus as described under Methods. Liver lysates were probed for SUMOylation levels with anti-SUMO1 (A) or anti-SUMO2/3 (B).

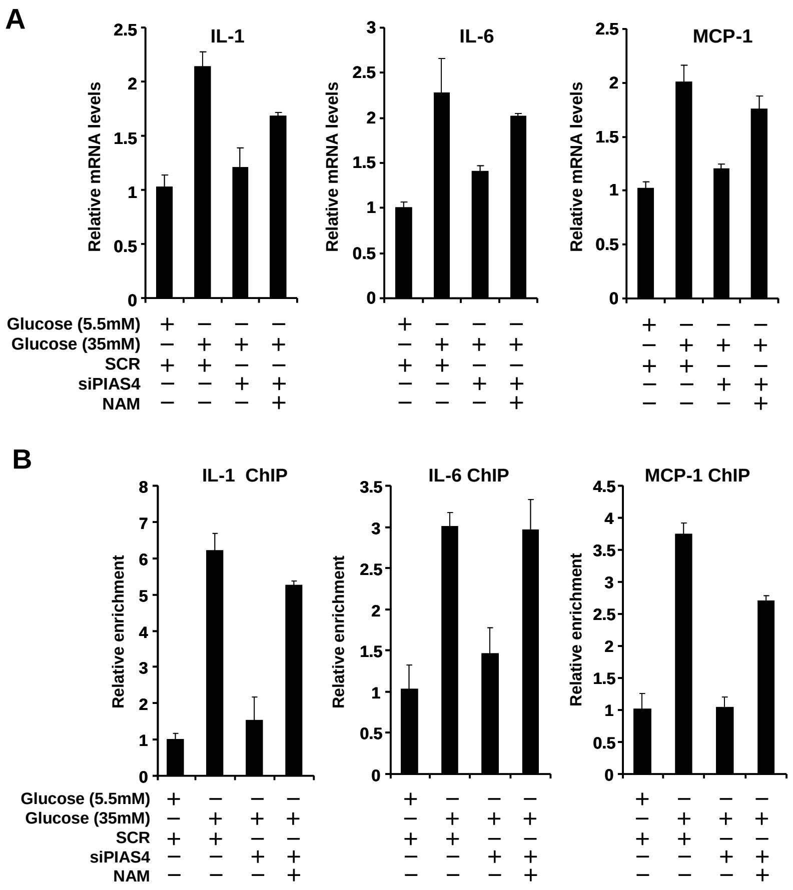

Fig.S4: (**A, B)** HepG2 cells were transfected with indicated siRNA followed by treatment with glucose and/or NAM. Expression levels of pro-inflammatory mediators were measured by qPCR (A). Binding of NF-κB/p65 to the promoters was examined by ChIP (B).

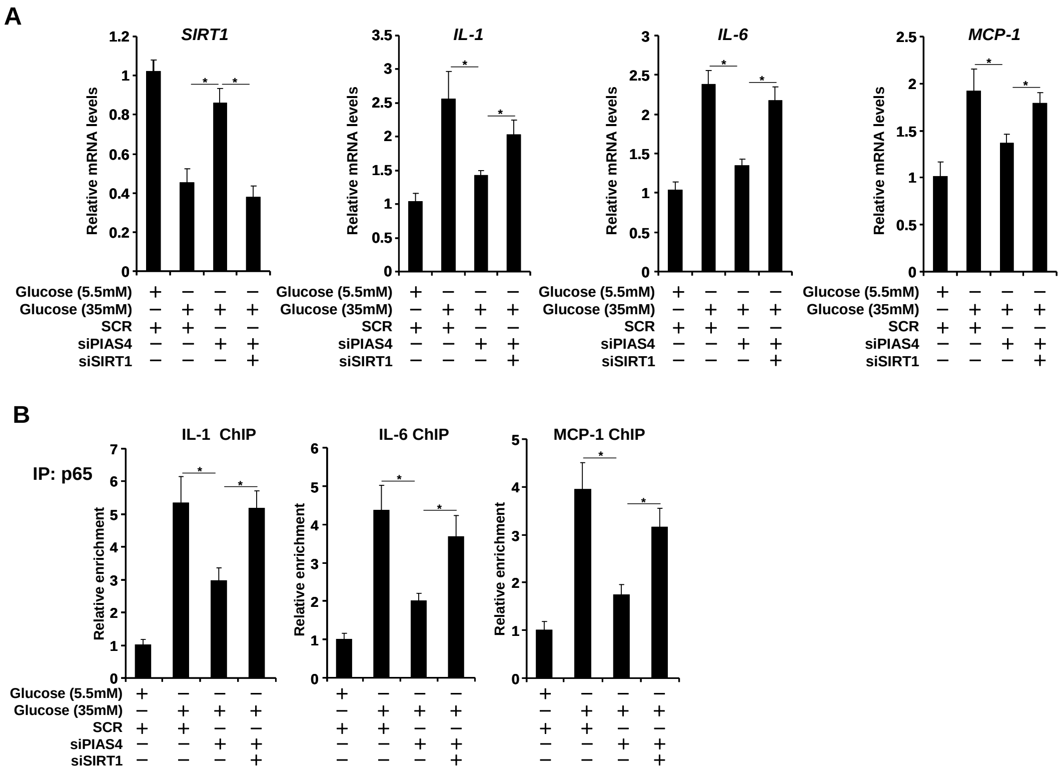

Fig.S5: (**A, B)** HepG2 cells were transfected with indicated siRNA followed by treatment with glucose. Expression levels of pro-inflammatory mediators were measured by qPCR (A). Binding of NF-κB/p65 to the promoters was examined by ChIP (B).
